# A Change of Publishers

**Published:** 1889-01

**Authors:** 


### A CHANGE OF PUBLISHERS.

With the beginning of the third volume the *Dental Review* will change publishers. Keener has no doubt found that dental journals are not a very profitable investment. H. D. Justi now assumes the responsibility of the publication, whether as an interested party, other than simply as publisher, deponent saith not. We did not, however, know that the well-known manufacturer of dental supplies had a publishing department in connection with his business before. The editor disclaims any sale or change of policy in the journal, and says that the *Review* will go on in the *old, old* way, as if no change had taken place.

